# In situ and split-thickness grafting for nail bed defects with bone exposure: A retrospective case series

**DOI:** 10.1097/MD.0000000000049822

**Published:** 2026-07-17

**Authors:** Chao Du, Xin Wang

**Affiliations:** a Department of Hand Surgery, Ningbo No.6 Hospital, Ningbo, Zhejiang Province, China; b Ningbo Clinical Research Center for Orthopedics, Sports Medicine & Rehabilitation, Ningbo, Zhejiang Province, China.

**Keywords:** autologous graft, bone exposure, case series, nail bed defect, reconstruction

## Abstract

Nail bed defects accompanied by bone exposure are challenging to treat because they rarely heal spontaneously and frequently require flap coverage or digit amputation. This study evaluated the postoperative function and appearance of nail bed defects repaired using in situ or split-thickness nail bed grafting. From March 2019 to December 2023, 28 (32 fingers) patients with nail bed defects and bone exposure underwent in situ or split-thickness autologous nail bed grafting. The clinical outcomes were quantitatively evaluated across five domains: nail plate appearance, proximal nail fold adherence, nail bed adherence, complications, and patient satisfaction. The postoperative follow-up was 12 months. Excellent and good postoperative effects were achieved for 90.6% of the fingers (29/32). Four complications or clinically important adverse-event cases were identified, comprising three score-defined poor outcomes and one additional good-outcome case with severe occupational dissatisfaction. One of the score-defined poor-outcome cases required a secondary outpatient procedure; no recurrent bone exposure, secondary flap coverage, or digital amputation occurred. Nail bed grafting may provide a useful reconstructive option for selected nail bed defects with bone exposure. In this preliminary case series, these techniques were straightforward and generally yielded favorable nail growth with a low rate of major complications; however, larger comparative studies are needed to validate these findings and refine clinical indications.

## 1. Introduction

Fingernails protect the soft tissues of the fingertips and play a crucial role in both digital aesthetics and functional activities. Nails aid in transmitting force and sensation, thus assisting in daily actions such as grasping, pinching, and clutching. Fingertip injuries are common in hand trauma and are often associated with fractures of the distal phalanx and nail bed disruption. Nail bed defects combined with bone exposure are challenging to treat because such wounds rarely heal spontaneously and often require amputation or flap surgery. Furthermore, because nail bed tissue possesses limited elasticity, direct primary closure to cover even small areas of exposed bone is technically difficult.^[1]^ Therefore, nail bed defects with exposed phalanges often require the selection of appropriate reconstructive materials. Coverage of such wounds with synthetic materials,^[2]^ autologous dermal grafting,^[3]^ or flap surgery^[4]^ frequently achieves wound coverage but fails to aid in forming a nail plate with a satisfactory cosmetic appearance. Relevant clinical and animal experimental studies have shown^[1,3,5]^ that early repair of nail bed defects can lead to the formation of a nail plate with satisfactory function and appearance, either via in situ grafting of the avulsed nail bed tissue or the use of split-thickness toe nail bed grafts, given the structural similarity between the toe and fingernail beds.

In this study, cases of nail bed defects with bone exposure treated with in situ grafting of the avulsed nail bed or autologous nail bed grafts were reviewed, and the indications, outcomes, and limitations of these methods were summarized.

## 2. Methods

### 2.1. Patients

**Figure 1.**
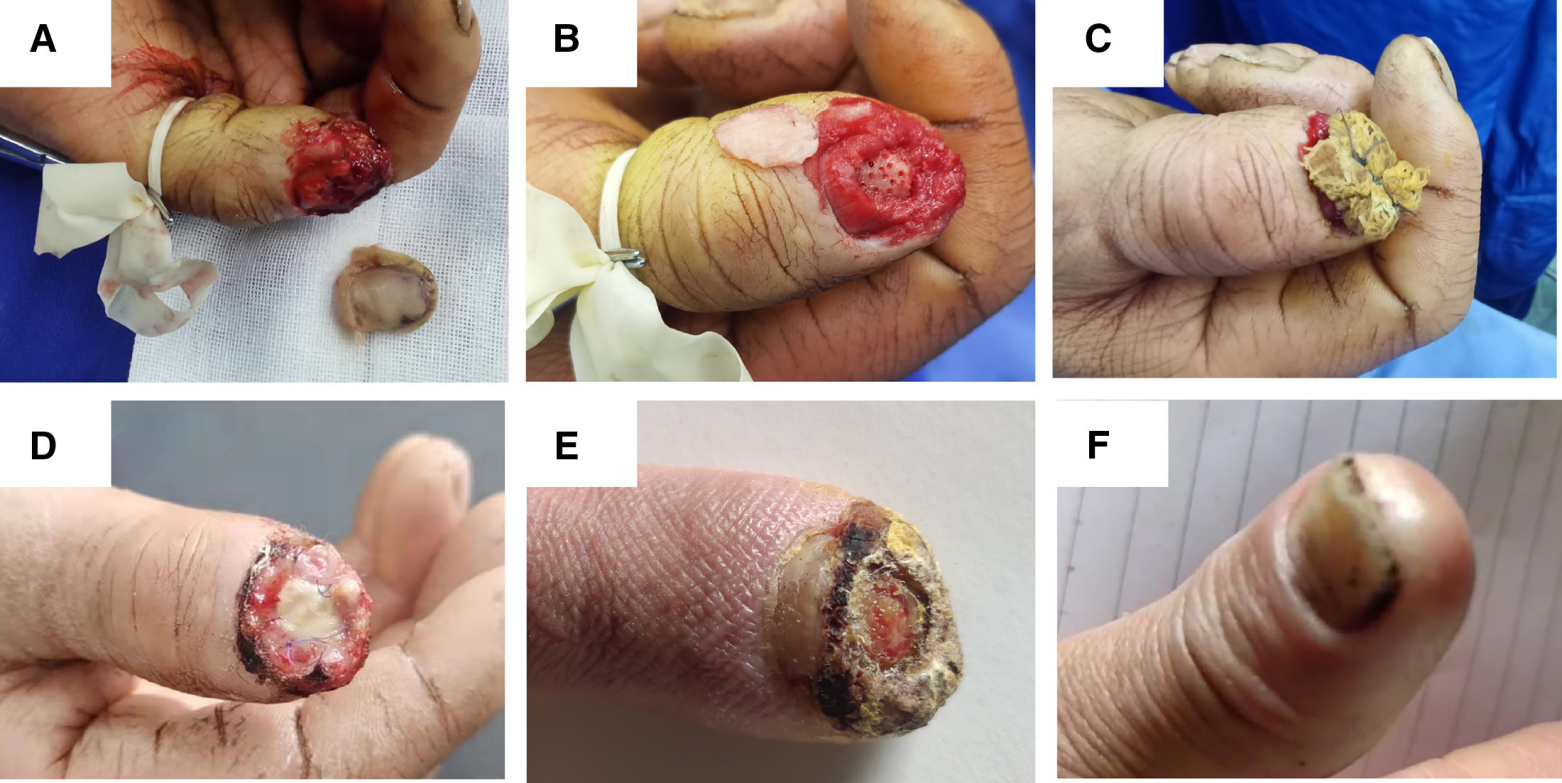
Representative case of in situ nail bed grafting for a nail bed defect with distal phalangeal bone exposure. (A) Preoperative appearance showing nail bed loss with bone exposure. (B) Intraoperative view showing recipient-site preparation with Kirschner-wire drilling and in situ nail bed grafting. (C) Immediate postoperative appearance. (D) Appearance at 2 weeks postoperatively. (E) Appearance at 3 months postoperatively. (F) Final outcome at 12 months postoperatively.

**Figure 2.**
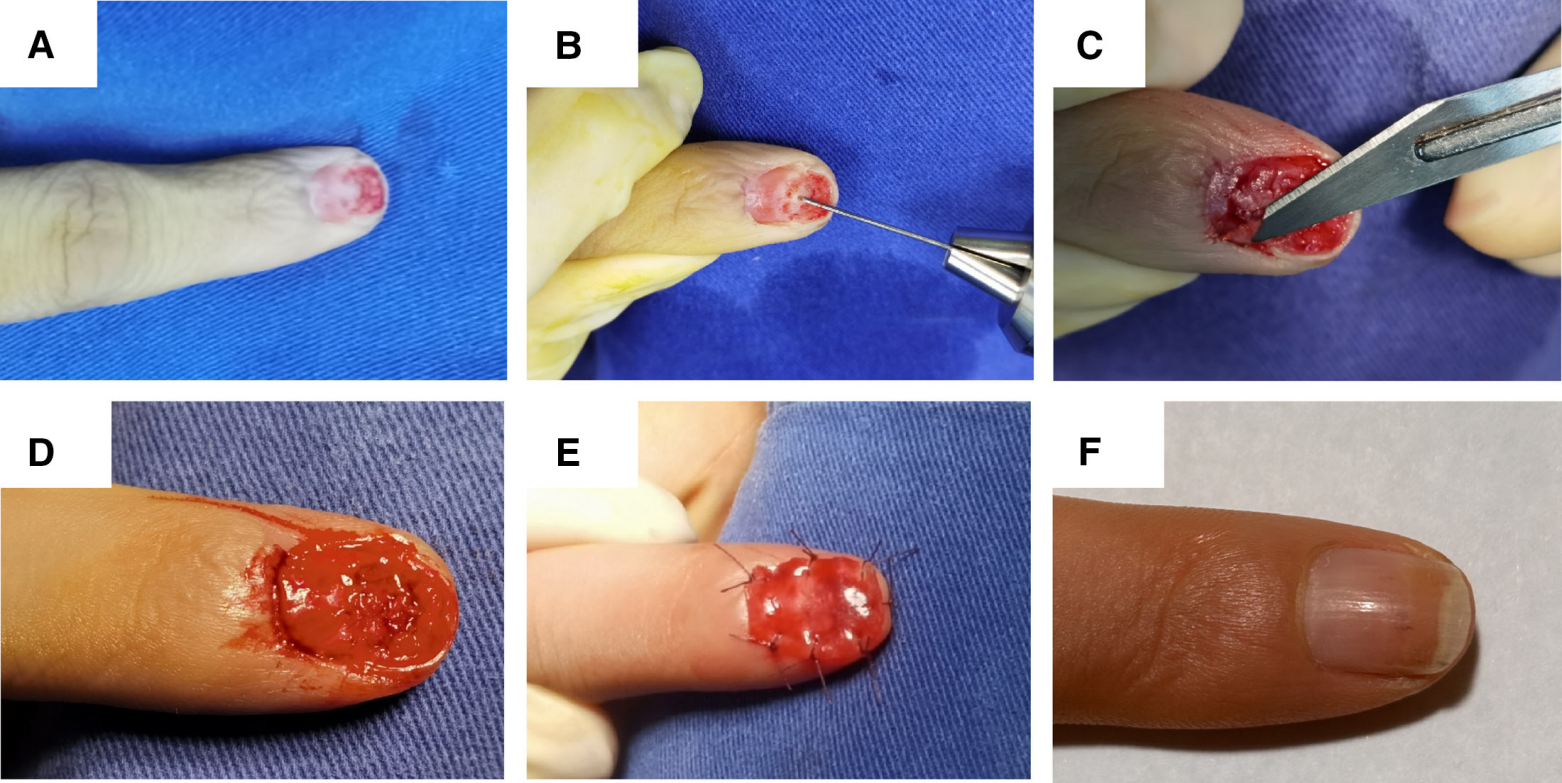
Representative case of same-finger split-thickness nail bed grafting for reconstruction of a nail bed defect with distal phalangeal bone exposure. (A) Preoperative appearance showing nail bed loss with bone exposure. (B) Recipient-site preparation with Kirschner-wire drilling. (C) Harvesting of a split-thickness nail bed graft from the same finger. (D) Graft fixation with 8-0 microsutures. (E) Completion of reconstruction with a moist dressing to maintain a moist wound environment. (F) Final outcome at 12 months postoperatively.

We performed a single-center retrospective analysis of a consecutive case series of patients undergoing surgery between March 2019 and December 2023. This study was approved by the Medical Ethics Committee of Ningbo No.6 Hospital, Ningbo, China (Ethics Approval No. 2023-28). All procedures were performed in accordance with the ethical standards of the institutional research committee and the Declaration of Helsinki. Given the retrospective nature of this study and the fully anonymized patient data, the requirement for active patient informed consent was formally waived by the Medical Ethics Committee. However, written informed consent for the publication of clinical photographs was explicitly obtained from the specific patients shown in Figures 1 and 2.

### 2.2. Indications

Inclusion criteria comprised patients over 16 years old with nail bed defects combined with bone exposure. The exclusion criteria were as follows: comminuted fractures of the distal phalanx with a bone defect measuring >5 mm in diameter; germinal matrix defects; soft tissue defects of the fingertips with a diameter measuring >10 mm or those with a high risk of necrosis; psychiatric diseases and cognitive abnormalities; and other severe systemic diseases that precluded surgical intervention.

### 2.3. Surgical procedure

All procedures were performed by experienced hand surgeons.

#### 2.3.1. In situ nail bed grafting

If the amputated nail bed was intact after trauma with minimal contamination, in situ nail bed grafting was performed (Fig. 1).

Anesthesia was achieved via a digital nerve block or brachial plexus block, and a rubber tourniquet was applied at the base of the digit to maintain a bloodless field. Next, the wound underwent thorough debridement, preserving as much of the exposed phalangeal periosteum as possible. The avulsed nail bed tissue to be grafted was meticulously trimmed, leveled, and soaked in povidone-iodine for at least 10 minutes. In cases involving distal phalangeal fracture, the fracture was reduced and fixed with 0.8-mm Kirschner wires. For smooth, exposed bone surfaces, a 0.8-mm Kirschner wire was used to drill punctate holes to create a bleeding recipient bed, whereas drilling was omitted if the bone surface was already comminuted or cancellous. Under microscope or loupe magnification, the trimmed and disinfected nail bed was grafted in situ using 8-0 absorbable sutures, and the surrounding eponychium or paronychium was secured with 5-0 absorbable interrupted sutures. Excess suture threads were retained to secure an iodophor-soaked cotton ball and Vaseline gauze pack over the graft for tie-over compression. If the defect was <4 mm and the original nail plate was available, it was also replaced over the graft as a biological stent. The nail plate was disinfected in povidone-iodine for over 10 minutes, perforated with 2 to 3 small drainage holes using a No. 15 blade, and secured with 5-0 absorbable sutures to prevent displacement. Postoperatively, a finger splint was used for fixation. Dressing changes were performed 2 to 3 days after surgery, and the tie-over dressings were removed at 2 weeks. For patients with internally fixated fractures, Kirschner wires were removed 4 to 6 weeks postoperatively upon radiographic confirmation of bone healing, while splint immobilization was maintained for an additional 2 to 4 weeks.

#### 2.3.2. Split-thickness nail bed grafting

If the avulsed nail bed was irreparable or unavailable, split-thickness nail bed grafting was performed using either a homodigital or heterodigital autograft. When the residual nail bed of the injured finger was sufficiently large, a split-thickness graft was harvested homodigitally from the uninjured portion of the same finger (Fig. 2). Otherwise, a heterodigital split-thickness graft was harvested from the great toe.

For both harvesting techniques, digital nerve block anesthesia was achieved, and a rubber tourniquet was applied to establish a bloodless surgical field. The graft was meticulously harvested using a scalpel at a precise depth where the blade was just translucent through the nail bed tissue. Owing to its larger surface area and minimal donor-site morbidity, the great toe was preferred when a homodigital graft was not feasible. Primary reconstruction was prioritized for all acute defects to maximize graft survival on a fresh recipient bed and prevent bone necrosis. In cases of severe wound contamination requiring staged secondary repair, emergency debridement was followed by temporary coverage of the exposed bone using a moist dressing before delayed split-thickness grafting.

### 2.4. Follow-up and assessment

Patients were followed up for 12 months postoperatively by the treating surgeon, who also performed the clinical outcome assessments using the predefined evaluation criteria. No patients were lost to follow-up.

Based on the formulas developed by Shepard,^[5]^ a quantitative scoring system (maximum 40 points) was implemented:

1. Nail plate appearance (maximum 10 points): Two points were deducted for each deformity, including unevenness, splitting, impaction, plate stacking, or other cosmetic defects.
2. Proximal nail fold (maximum 5 points): No score was given in cases of attachment to the nail bed or pterygium hyperplasia.
3. Attachment of the nail plate and the nail bed (maximum 5 points): No score was given if the separation of the nail bed from the nail plate was >1/3 of the total nail bed surface area.
4. Complications (maximum 10 points): Five points were deducted for persistent discomfort or pain.
5. Patient satisfaction (maximum 10 points): The score was obtained using a follow-up questionnaire.

Qualitatively, the total scores were categorized as follows: excellent, ≥35 points; good, ≥30 and <35 points; and poor, <30 points.

### 2.5. Statistical analysis

Descriptive statistics were used to summarize patient characteristics and clinical outcomes. Continuous variables are presented as mean ± standard deviation or median (range), as appropriate. Categorical variables are reported as frequencies and percentages.

Because of the limited sample size and the markedly unequal distribution among treatment subgroups, formal comparative statistical analyses were considered underpowered and potentially misleading. Therefore, outcomes were primarily analyzed descriptively. Subgroup results according to reconstructive technique are presented to facilitate the comparison of clinical outcomes among treatment categories.

Statistical analyses were performed using the Statistical Package for the Social Sciences version 26.0 (IBM Corp.).

This study was reported in accordance with the Strengthening the Reporting of Observational Studies in Epidemiology statement.

## 3. Results

### 3.1. Baseline characteristics

**Figure 3.**
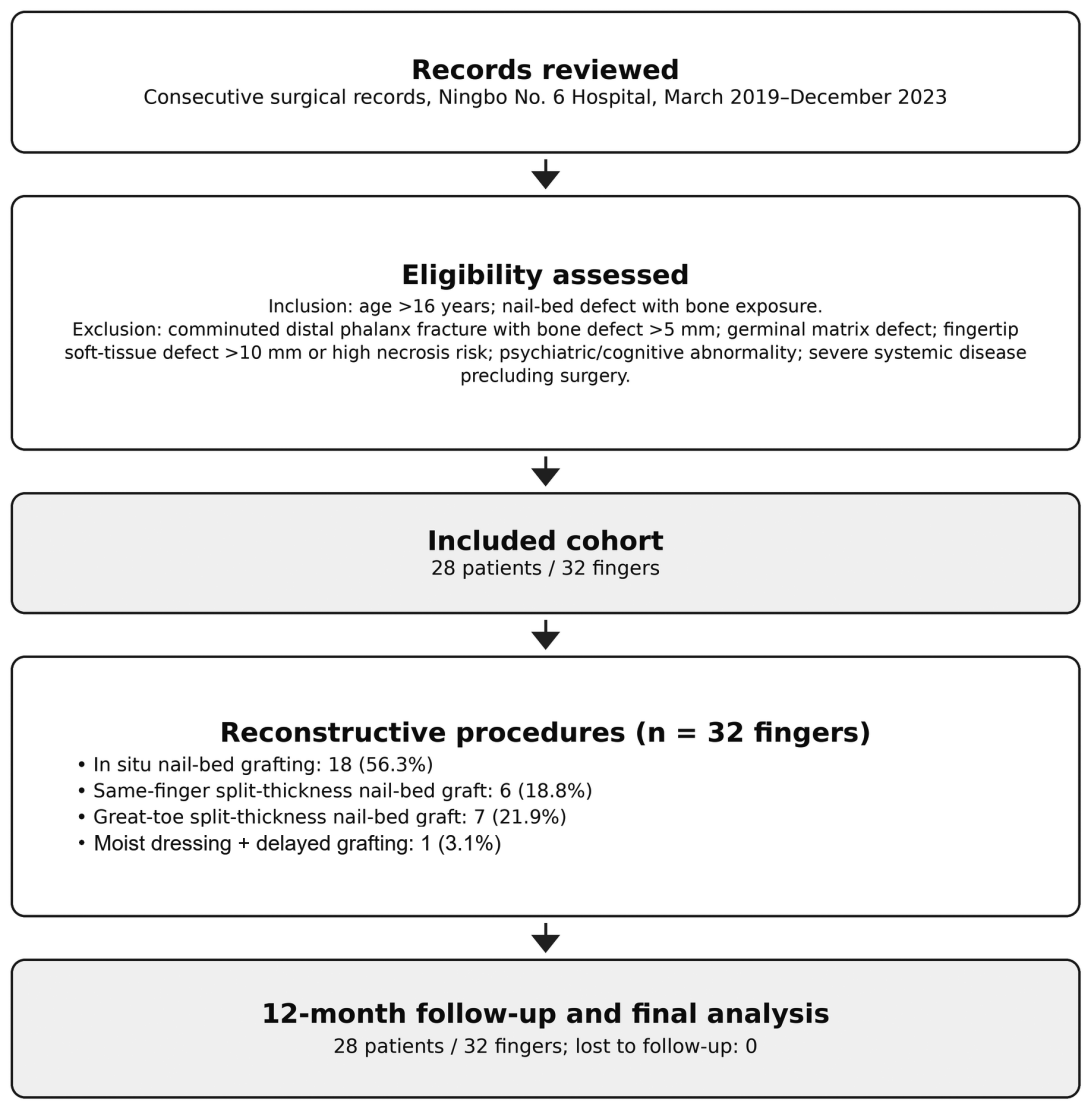
Flow diagram of patient selection and follow-up.

During the study period, 28 (32 fingers) patients were finally included for retrospective outcome assessment after rigorous eligibility screening (Fig. 3). The mean age was 37.5 ± 14.3 years (range: 16–67 years). Twenty (71.4%) patients were male and eight (28.6%) were female. The mean diameter of bone exposure was 4.1 ± 1.2 mm (range: 2–7 mm). Baseline demographic and injury characteristics are summarized in Table 1. Detailed baseline characteristics are presented in Table S1, Supplemental Digital Content 1.

**Table 1:** Baseline characteristics of the study cohort (28 patients, 32 digits).

Characteristic	Value
Age (yr), mean ± SD	37.5 ± 14.3
Male sex, n (%)	20 (71.4)
Bone exposure diameter (mm), mean ± SD	4.1 ± 1.2
Fracture, n (%)	10 (31.3)
Proximal nail fold injury, n (%)	9 (28.1)

Age and sex were calculated per patient (n = 28). Injury characteristics were calculated per digit (n = 32).

n = number, SD = standard deviation.

### 3.2. Surgical procedures

A total of 32 injured fingers underwent reconstruction using four different techniques according to the condition of the residual nail bed and the extent of tissue loss. In situ nail bed grafting was the most frequently performed procedure, accounting for 18 (56.3%) fingers. Same-finger split-thickness nail bed grafting was performed in six (18.8%) fingers, while great toe split-thickness nail bed grafting was used in seven (21.9%) fingers. One (3.1%) finger was reconstructed using moist dressing followed by delayed nail bed grafting. The distribution of surgical procedures is summarized in Table 2.

**Table 2:** Surgical procedures performed.

Procedure	n (%)
In situ nail bed grafting	18 (56.3)
Same-finger split-thickness nail bed graft	6 (18.8)
Great toe split-thickness nail bed graft	7 (21.9)
Moist dressing + delayed grafting	1 (3.1)

Percentages were calculated per digit (n = 32).

n = number.

### 3.3. Outcomes according to the reconstructive technique

Four complications or clinically important adverse-event cases were identified: three score-defined poor outcomes and one additional good-outcome case with severe occupational dissatisfaction. One patient required a secondary outpatient procedure for paronychia. No donor-site complications, recurrent bone exposure, secondary flap coverage, or digital amputation occurred. Score-defined outcomes according to the reconstructive technique are presented in Table 3, while the complication/adverse-event cases are described below and detailed in Table S2, Supplemental Digital Content 2.

**Table 3:** Descriptive outcomes according to reconstructive technique.

Surgical technique	No. of fingers	Mean score ± SD (or score for n = 1)	Excellent/good, n (%)	Poor, n (%)
In situ nail bed grafting	18	37.4 ± 3.2	17 (94.4%)	1 (5.6%)
Same-finger split-thickness nail bed graft	6	35.7 ± 4.0	5 (83.3%)	1 (16.7%)
Great toe split-thickness nail bed graft	7	34.9 ± 5.0	6 (85.7%)	1 (14.3%)
Moist dressing + delayed grafting	1	40.0	1 (100%)	0
Total	32	36.6 ± 3.8	29 (90.6%)	3 (9.4%)

Percentages were calculated within each reconstructive technique subgroup.

n = number, SD = standard deviation.

In situ nail bed grafting was the most commonly performed procedure (18 fingers, 56.3%) and achieved the highest mean outcome score among the techniques with more than one case (37.4 ± 3.2), with 94.4% of these fingers achieving excellent or good outcomes. Same-finger split-thickness nail bed grafting and great toe split-thickness nail bed grafting yielded mean scores of 35.7 ± 4.0 and 34.9 ± 5.0, respectively, with favorable outcomes observed in most patients. The single patient treated with moist dressing followed by delayed nail bed grafting achieved an excellent outcome with a score of 40 points. Overall, 29 of 32 fingers (90.6%) achieved excellent or good outcomes, whereas three (9.4%) fingers were classified as having poor outcomes. Individual postoperative outcome scores are shown in Table S2, Supplemental Digital Content 2. Owing to the limited sample size and unequal distribution among treatment groups, formal comparative statistical analyses were not performed.

### 3.4. Complications and poor outcomes

Complications or clinically important adverse events were noted in four fingers, while three fingers were classified as score-defined poor outcomes. In the first poor-outcome case, although the exposed bone diameter was only 2 mm, the injury was located in close proximity to the germinal matrix; this adjacent tissue damage ultimately caused nail impaction and persistent postoperative hyperesthesia. In the second poor-outcome case, a severe preoperative injury to the proximal nail fold led to marked pterygium hyperplasia and subsequent secondary paronychia. This was the only case requiring a secondary outpatient procedure, which consisted of a simple incision, debridement, and topical application of an iodophor-soaked dressing. In the third poor-outcome case, despite achieving adequate wound coverage, the patient exhibited secondary nail deformity; although functionally stable, the aesthetic result initially displeased the patient, who eventually accepted the outcome. The fourth complication/adverse-event case involved a tannery worker who achieved a total score of 34 points and was therefore classified as having a good rather than a poor outcome. However, his mild residual fingertip discomfort and hypersensitivity were amplified by frequent occupational exposure to irritating fluids and high physical demands, leading to substantial dissatisfaction and a retrospective preference for primary digital amputation over reconstruction. Individual postoperative outcome scores are shown in Table S2, Supplemental Digital Content 2.

## 4. Discussion

Nail bed injuries can be reconstructed using various modalities, including autologous full-thickness skin grafts,^[3,6]^ autologous split-thickness skin grafts,^[7]^ synthetic materials,^[2,8–10]^ autologous split-thickness nail bed grafts,^[1,5,11–13]^ autologous full-thickness nail bed grafts,^[7,13]^ regional fascial flaps,^[4,14,15]^ and microsurgical nail flap transfer from the great toe.^[16,17]^ The nail bed plays an important role in the formation of the nail plate. However, when defects are covered with nonungual tissues, the subsequent nail plate frequently exhibits severe deformity or growth arrest. Full-thickness nail bed grafts often result in severe nail deformity at the donor site. Therefore, split-thickness nail bed grafts have gradually become the first-line therapeutic strategy for nail bed repair.^[1,13]^

Currently, a universally accepted classification standard^[5]^ for nail bed injuries remains unavailable, with most historical grading systems relying on variables such as defect surface area, concomitant distal phalangeal fractures, germinal matrix or proximal nail fold involvement, and associated fingertip soft tissue loss. Although bone exposure areas of <2 mm may heal spontaneously via secondary intention, such conservative management should be restricted to patients with low aesthetic expectations. If favorable function and appearance are desired, primary reconstruction is the preferred treatment. In patients with severe damage to the distal phalanx, nail bed repair is futile because the shape of the nail is largely determined by the underlying phalanx, which provides the anatomical support. Therefore, in this study, fractures of the distal phalanx with bone defects >5 mm in diameter were excluded. Furthermore, since substantial germinal matrix defects often necessitate complex microsurgical nail flap transfer rather than simple grafting, patients with extensive germinal matrix damage were excluded from our cohort; however, in clinical practice, a germinal matrix defect of <2 mm with a repairable nail bed can still yield a satisfactory nail plate following meticulous suturing. Patients with larger soft tissue defects or those at a greater risk of necrosis are also not suitable for nail bed repair as inadequate soft-tissue coverage predisposes the patient to chronic pain, hypersensitivity, and hook-nail deformities.

Evaluation of function and appearance after nail bed injury still depends on empirical assessments. Based on the evaluation indicators proposed by Shepard^[5]^, we utilized a modified scoring system that reallocates weights across critical domains and, importantly, integrates patient-reported satisfaction as a major component. However, as illustrated by the tannery-worker case, occupational exposure and work-related functional demands may strongly influence satisfaction without necessarily lowering the composite score into the poor-outcome category. This limitation highlights the need for more standardized, comprehensive, and context-sensitive clinical tools. Quantitative assessments and evidence-based treatment remain important future research directions for nail bed repair.

More often than not, nail bed repair surgery is perceived primarily as a form of cosmetic restoration. If there is a high risk of postoperative fingertip discomfort or if the patient has low aesthetic requirements, surgery should be approached with caution. The complication and poor-outcome cases in this study highlight specific clinical challenges. These findings suggest that patients with severe proximal nail fold injury, injuries near the germinal matrix, or high occupational demands, such as the tannery worker in our series, may require careful preoperative counseling and consideration of alternative reconstructive options.

This study has several limitations. First, the sample size was relatively small, and the study was conducted at a single institution, which may limit the generalizability of the findings. Furthermore, strict eligibility criteria were applied because nail bed reconstruction is primarily considered a cosmetic and function-preserving procedure for highly motivated patients. Consequently, some potentially suitable patients may have been excluded, introducing selection bias. Second, this study lacked a control or comparison group, making it difficult to directly compare these techniques against alternative treatment strategies, such as flap reconstruction or conservative management. Furthermore, several reconstructive techniques – including in situ nail bed grafting, homodigital split-thickness grafting, heterodigital great toe grafting, and moist dressing followed by delayed nail bed grafting – were analyzed within the same cohort. Owing to the limited sample size and unequal distribution among subgroups, meaningful comparative analyses could not be performed, and the relative advantages of each technique remain to be determined. Third, and importantly, outcome assessment was performed solely by the treating surgeon using a modified Shepard scoring system. Although this scoring system has been widely used for postoperative evaluation, its weighting has not been formally validated. Because the evaluations relied on a single investigator, intra- or inter-rater reliability could not be established. Moreover, outcome assessment was not blinded to the surgical technique, which may have introduced observer bias. Finally, detailed subgroup analyses according to injury mechanism, defect topography, and occupational demands were not performed. Future prospective, multicenter studies with larger cohorts, standardized outcome measures, blinded assessment, and appropriate comparative groups are needed to further validate these preliminary findings and refine the indications for each reconstructive technique.

In summary, the findings of this retrospective case series demonstrate that in situ nail bed grafting and split-thickness nail bed grafting may be useful options for the primary reconstruction of selected nail bed defects with limited bone exposure. Favorable functional and aesthetic outcomes were achieved in most patients, and no recurrent bone exposure, secondary flap coverage, or digital amputation occurred. When applied in carefully selected cases, these techniques offer simple and practical approaches to preserve the specialized ungual architecture without resorting to complex flap surgery. Nevertheless, further prospective studies with larger cohorts and comparative designs are warranted to validate these preliminary findings and refine specific clinical indications.

## Acknowledgments

We sincerely acknowledge all the study participants and the clinical staff for their valuable contribution and support in the successful conduct of this study.

## Author contributions

**Data curation:** Chao Du.

**Formal analysis:** Chao Du.

**Investigation:** Chao Du.

**Methodology:** Chao Du.

**Project administration:** Chao Du.

**Resources:** Chao Du.

**Software:** Chao Du.

**Supervision:** Chao Du.

**Validation:** Chao Du.

**Visualization:** Chao Du.

**Conceptualization:** Xin Wang.

**Funding acquisition:** Xin Wang.

**Writing – original draft:** Chao Du.

**Writing – review & editing:** Chao Du.




