# Supplementary material for: In situ and split-thickness grafting for nail bed defects with bone exposure: A retrospective case series
Source: Medicine (Baltimore). 2026 Jul 17;105(29):e49822. doi: 10.1097/MD.0000000000049822 (PMC13384654; doi:10.1097/MD.0000000000049822)
Supplement: Supplementary file 1 [file medi-105-e49822-s001.docx]

**Supplementary Table 1. Detailed baseline characteristics of all included digits.**

| **Patient/digit number** | **Sex** | **Age, y** | **Finger** | **Bone exposure diameter, mm** | **Fracture status** | **Proximal nail fold injury** | **Surgical method** |
| --- | --- | --- | --- | --- | --- | --- | --- |
| 1 | Male | 24 | L2 | 3 | 0 | Yes | 1 |
| 2 | Male | 27 | R1 | 5 | 1 | No | 1 |
| 3 (1) | Female | 32 | R1 | 4 | 0 | No | 2 |
| 3 (2) | Female | 32 | R2 | 3 | 2 | Yes | 1 |
| 4 | Male | 45 | L2 | 4 | 0 | No | 1 |
| 5 | Male | 55 | L1 | 3 | 0 | No | 1 |
| 6 | Female | 19 | R5 | 2 | 0 | No | 2 |
| 7 | Male | 48 | R1 | 4 | 1 | No | 1 |
| 8 | Male | 31 | R1 | 6 | 2 | Yes | 3 |
| 9 | Female | 44 | L1 | 3 | 0 | Yes | 1 |
| 10 | Female | 52 | L2 | 3 | 0 | No | 4 |
| 11 | Male | 16 | R1 | 6 | 2 | No | 3 |
| 12 | Male | 43 | L2 | 4 | 0 | No | 1 |
| 13 | Female | 23 | R3 | 4 | 1 | No | 3 |
| 14 | Male | 56 | R3 | 3 | 0 | Yes | 1 |
| 15 | Male | 47 | R1 | 7 | 1 | No | 1 |
| 16 | Male | 33 | R3 | 5 | 0 | Yes | 1 |
| 17 | Male | 21 | R3 | 4 | 0 | No | 3 |
| 18 | Female | 23 | L1 | 5 | 0 | No | 3 |
| 19 | Male | 60 | L2 | 3 | 0 | No | 1 |
| 20 | Male | 31 | L1 | 6 | 1 | No | 3 |
| 21 | Female | 19 | L5 | 3 | 0 | No | 3 |
| 22 | Male | 34 | L1 | 6 | 1 | Yes | 1 |
| 23(1) | Female | 21 | R1 | 3 | 0 | No | 2 |
| 23(2) | Female | 21 | R2 | 3 | 0 | No | 1 |
| 23(3) | Female | 21 | L1 | 4 | 0 | No | 2 |
| 24 | Male | 37 | R1 | 5 | 0 | Yes | 1 |
| 25 | Male | 67 | L4 | 3 | 2 | No | 2 |
| 26 | Male | 38 | L1 | 5 | 0 | Yes | 1 |
| 27(1) | Male | 55 | R2 | 4 | 0 | No | 1 |
| 27(2) | Male | 55 | L2 | 3 | 0 | No | 2 |
| 28 | Male | 49 | R2 | 4 | 0 | No | 1 |

**Note:** For patients with multiple affected digits, each digit was treated as a separate observational unit and labeled using numbers in parentheses. For example, 3(1) and 3(2) represent two different digits from Patient 3.

Finger designation was recorded as follows: 1 = thumb, 2 = index finger, 3 = middle finger, 4 = ring finger, and 5 = little finger.

Fracture status: 0, no fracture; 1, stable fracture or fracture reducible with internal fixation; 2, comminuted fracture without bone defects greater than 5 mm.

Surgical method: 1, in situ nail bed grafting; 2, same-finger split-thickness nail bed grafting; 3, great toe split-thickness nail bed grafting; 4, moist dressing coverage followed by delayed nail bed grafting.

**Abbreviations:** L, left; mm, millimeter; R, right; y, years.
