# Supplementary material for: In situ and split-thickness grafting for nail bed defects with bone exposure: A retrospective case series
Source: Medicine (Baltimore). 2026 Jul 17;105(29):e49822. doi: 10.1097/MD.0000000000049822 (PMC13384654; doi:10.1097/MD.0000000000049822)
Supplement: Supplementary file 2 [file medi-105-e49822-s002.docx]

**Supplementary Table 2. Individual postoperative outcome scores.**

| **Patient/digit number** | **Appearance** | **Proximal nail fold** | **Nail plate-nail bed attachment** | **Complications** | **Satisfaction** | **Quantitative score** |
| --- | --- | --- | --- | --- | --- | --- |
| 1 | 10 | 5 | 5 | 10 | 9 | 39 |
| 2 | 8 | 5 | 5 | 10 | 10 | 38 |
| 3(1) | 10 | 5 | 5 | 10 | 10 | 40 |
| 3(2) | 10 | 5 | 5 | 10 | 10 | 40 |
| 4 | 10 | 5 | 5 | 10 | 10 | 40 |
| 5 | 10 | 5 | 5 | 10 | 10 | 40 |
| 6 | 8 | 5 | 5 | 5 | 6 | 29 |
| 7 | 10 | 5 | 5 | 10 | 8 | 38 |
| 8 | 8 | 5 | 5 | 0 | 6 | 24 |
| 9 | 10 | 5 | 5 | 10 | 10 | 40 |
| 10 | 10 | 5 | 5 | 10 | 10 | 40 |
| 11 | 8 | 5 | 5 | 10 | 8 | 36 |
| 12 | 10 | 5 | 5 | 10 | 8 | 38 |
| 13 | 10 | 5 | 5 | 10 | 9 | 39 |
| 14 | 10 | 0 | 5 | 10 | 8 | 33 |
| 15 | 10 | 5 | 5 | 10 | 10 | 40 |
| 16 | 10 | 5 | 5 | 10 | 10 | 40 |
| 17 | 8 | 5 | 5 | 10 | 7 | 35 |
| 18 | 10 | 5 | 5 | 10 | 7 | 37 |
| 19 | 8 | 5 | 5 | 10 | 8 | 36 |
| 20 | 10 | 5 | 5 | 10 | 8 | 38 |
| 21 | 8 | 5 | 5 | 10 | 5 | 35 |
| 22 | 8 | 0 | 5 | 10 | 6 | 29 |
| 23(1) | 10 | 5 | 5 | 10 | 7 | 37 |
| 23(2) | 10 | 5 | 5 | 10 | 7 | 37 |
| 23(3) | 10 | 5 | 5 | 10 | 7 | 37 |
| 24 | 10 | 5 | 5 | 10 | 4 | 34 |
| 25 | 10 | 5 | 0 | 10 | 8 | 33 |
| 26 | 10 | 5 | 5 | 10 | 9 | 39 |
| 27(1) | 10 | 5 | 5 | 5 | 8 | 33 |
| 27(2) | 10 | 5 | 5 | 10 | 8 | 38 |
| 28 | 10 | 5 | 5 | 10 | 9 | 39 |

**Note:** For patients with multiple affected digits, each digit was treated as a separate observational unit and labeled using numbers in parentheses. For example, 3(1) and 3(2) represent two different digits from Patient 3.

Complications or clinically important adverse events were noted in four fingers, while three fingers met the score-defined poor-outcome threshold. In Patient 6, although the exposed bone diameter was only 2 mm, the injury was located in close proximity to the germinal matrix; this adjacent tissue damage ultimately caused nail impaction and persistent postoperative hyperesthesia. In Patient 8, a severe preoperative injury to the proximal nail fold led to marked pterygium hyperplasia and subsequent secondary paronychia; this was the only case requiring a secondary outpatient procedure, consisting of simple incision, debridement, and topical application of an iodophor-soaked dressing. Patient 22, despite achieving adequate wound coverage, exhibited secondary nail deformity; although functionally stable, the aesthetic result initially displeased the patient, who eventually accepted the outcome. Patient 24 achieved a radiographically and structurally favorable recovery and had a total score of 34 points, which remained in the good-outcome category. However, this patient was employed in a tannery, requiring frequent exposure to irritating fluids and high physical demands. Mild residual fingertip discomfort and hypersensitivity substantially interfered with his occupational duties, leading to severe dissatisfaction and a retrospective preference for primary digital amputation over reconstruction. This case was therefore counted as a complication/adverse-event case but not as a score-defined poor outcome.
